# Rhizobacterial colonization of roots modulates plant volatile emission and enhances the attraction of a parasitoid wasp to host-infested plants

**DOI:** 10.1007/s00442-015-3277-7

**Published:** 2015-03-19

**Authors:** Nurmi Pangesti, Berhane T. Weldegergis, Benjamin Langendorf, Joop J. A. van Loon, Marcel Dicke, Ana Pineda

**Affiliations:** Laboratory of Entomology, P.O. Box 8031, 6700 EH Wageningen, The Netherlands

**Keywords:** HIPVs, Indirect defense, *Microplitis mediator*, Parasitoid behavior, Beneficial microbes

## Abstract

Beneficial root-associated microbes modify the physiological status of their host plants and affect direct and indirect plant defense against insect herbivores. While the effects of these microbes on direct plant defense against insect herbivores are well described, knowledge of the effect of the microbes on indirect plant defense against insect herbivores is still limited. In this study, we evaluate the role of the rhizobacterium *Pseudomonas fluorescens* WCS417r in indirect plant defense against the generalist leaf-chewing insect *Mamestra brassicae* through a combination of behavioral, chemical, and gene-transcriptional approaches. We show that rhizobacterial colonization of *Arabidopsis thaliana* roots results in an increased attraction of the parasitoid *Microplitis mediator* to caterpillar-infested plants. Volatile analysis revealed that rhizobacterial colonization suppressed the emission of the terpene (*E*)-α-bergamotene and the aromatics methyl salicylate and lilial in response to caterpillar feeding. Rhizobacterial colonization decreased the caterpillar-induced transcription of the terpene synthase genes *TPS03* and *TPS04*. Rhizobacteria enhanced both the growth and the indirect defense of plants under caterpillar attack. This study shows that rhizobacteria have a high potential to enhance the biocontrol of leaf-chewing herbivores based on enhanced attraction of parasitoids.

**Electronic supplementary material:**

The online version of this article (doi:10.1007/s00442-015-3277-7) contains supplementary material, which is available to authorized users.

## Introduction

Plants are exposed to attack by various insect herbivores and defend themselves directly, e.g., by producing toxic compounds, and indirectly, e.g., by emitting herbivore-induced plant volatiles (HIPVs) that attract natural enemies of the herbivores (Clavijo McCormick et al. 2012; Dicke and Baldwin 2010; Jensen et al. 2002; Turlings et al. 1995). The effect of herbivore-induced plant volatiles (HIPVs) on the behavior of natural enemies has been widely studied in the context of interactions between one plant, one insect herbivore, and one natural enemy (Mumm and Dicke 2010). The exploration of such tritrophic interactions is now being extended to interactions in more complex systems (Dicke et al. 2009; Heil 2014; Pineda et al. 2013). For instance, insect eggs, multiple insect herbivores, pathogenic and beneficial microbes, and belowground herbivores have been shown to affect indirect plant defenses (Fatouros et al. 2012; Ponzio et al. 2014; Rasmann and Turlings 2007; Reymond 2013; Soler et al. 2007; Dam and Heil 2011; Zhang et al. 2013). Belowground beneficial microbes such as mycorrhizae, rhizobia, and rhizobacteria constitute a fascinating functional group in the plant-associated community that can enhance plant growth and resistance against pathogens and herbivorous insects (Hartley and Gange 2009; Pineda et al. 2010; Pozo and Azcon-Aguilar 2007). However, more recently, the effects of mutualistic microbes on the emission of HIPV and on natural enemies of herbivorous insects have been studied (Ballhorn et al. 2013; Pineda et al. 2013; Schausberger et al. 2012).

Root-associated microbes modify plant physiology and can therefore have an impact on direct and indirect plant defense against insects. In the context of indirect plant defense, root-colonizing microbes have been shown to be beneficial to the plant by enhancing the attraction or performance of natural enemies of the herbivores through plant-mediated effects (Gange et al. 2003; Guerrieri et al. 2004; Hempel et al. 2009; Hoffmann et al. 2011; Schausberger et al. 2012). Interestingly, the effect of beneficial microbes on the emission of HIPVs varies, from increased emission of the terpenoids β-ocimene and β-caryophyllene or HIPVs in general (Pineda et al. 2013) to suppressed emission of HIPVs (Fontana et al. 2009). However, experimental evidence showed that increased emission of HIPVs induced by beneficial microbes has differential effects on the attractiveness to the natural enemies of the herbivore, ranging from increased attractiveness (Schausberger et al. 2012) to repellence (Pineda et al. 2013).

Synthesis of plant secondary metabolites and HIPVs involved in direct and indirect plant defense is regulated by interconnected phytohormonal signaling pathways. The plant hormones jasmonic acid (JA), ethylene (ET), and salicylic acid (SA) are the main phytohormones regulating those herbivore-induced responses in the plant (Dicke and Poecke 2002; Kessler and Baldwin 2002; Pieterse et al. 2012). In the context of indirect defense, depending on the species and feeding mode of the insect herbivores, different combinations of hormonal signaling pathways can be induced, resulting in the synthesis of specific blends of HIPVs that attract natural enemies of the herbivores (Heil 2014; Wei et al. 2014; Zhang et al. 2013). The plant hormone JA regulates the synthesis of VOCs such as green leaf volatiles (GLVs) and terpenoids, whereas SA regulates the shikimate pathway and the emission of volatiles such as methyl salicylate (MeSA) (Dicke and Poecke 2002; Maffei et al. 2011; Poecke and Dicke 2002). Several beneficial microbes, such as the well-studied rhizobacterium *Pseudomonas fluorescens* WCS417r, are known to modulate JA and ET signaling (Ent et al. 2009; Wees et al. 2008), leading to enhanced expression of defense-associated genes and to modification of the plant’s response to insect herbivores from different feeding guilds (Pangesti et al. 2015; Pineda et al. 2012; Oosten et al. 2008). In contrast, the effects of root colonization by beneficial microbes on indirect defenses and natural enemies of the herbivores are still largely unknown.

In the study reported in the present paper, we aimed to evaluate the role of *P. fluorescens* WCS417r in plant indirect defense upon feeding by the herbivore *Mamestra brassicae* by evaluating behavioral choices and the performance of the parasitic wasp *Microplitis mediator*. This parasitoid is a generalist solitary larval endoparasitoid that parasitizes first- to third-larval instars of *M. brassicae* (Malcicka and Harvey 2014). *Microplitis mediator* is one of the most important natural enemies of the generalist herbivore *M. brassicae* (Lauro et al. 2005), and is known to parasitize ca. 40 species of lepidopteran herbivores (Li et al. 2006a, b). The rhizobacterium *P. fluorescens* WCS417r is known to enhance plant resistance via a mechanism called induced systemic resistance (ISR), which is effective against pathogenic microbes and insect herbivores; furthermore, the rhizobacterium is able to promote plant growth (Pangesti et al. 2015; Pieterse et al. 1998; Oosten et al. 2008; Wees et al. 2008; Zamioudis et al. 2013). By combining behavioral, chemical, and transcriptional approaches, we tested the hypotheses that rhizobacteria-treated plants will (1) be more attractive to the parasitic wasp *M. mediator* upon caterpillar herbivory and support better performance of the parasitoid; (2) emit higher amounts of VOCs upon caterpillar herbivory; (3) increase the expression of two terpene synthase genes: *TPS03*, encoding (*E*,*E*)-α-farnesene synthase, an enzyme involved in the biosynthesis of (*E*,*E*)-α-farnesene (Huang et al. 2010); and *TPS04*, which encodes geranyllinalool synthase (GES), a major enzyme involved in 4,8,12-trimethyltrideca-1,3,7,11-tetraene [(*E*,*E*)-TMTT] biosynthesis (Herde et al. 2008) upon feeding by caterpillar herbivory.

## Materials and methods

### Plant growth and insect rearing

Seeds of *Arabidopsis thaliana* Col-0 were sown in sand (masonry sand, Van Leusden B.V., P.A. Klundert, The Netherlands). Seedlings (10 days old) were transplanted into pots (120 ml) containing a 1:1 (v/v) mixture of potting soil:sand. In this study, we used commercial potting soil for *Arabidopsis* (Lentse Arabidopsis-grond, Lent, The Netherlands). Plant growth conditions have been described in Pangesti et al. (2015). Once a week, 10 ml of a half-strength Hoagland solution/pot (Sigma–Aldrich, St. Louis, MO, USA) was added (Oosten et al. 2008). In all experiments, 5- to 6-week-old plants in the vegetative stage were used.

The generalist insect herbivore *M. brassicae* L. (Lepidoptera: Noctuidae; Cabbage moth) was reared on *Brassica oleracea* L. var. *gemmifera* cv. Cyrus (Brussels sprouts) in a climate chamber (22 ± 2 °C, 40–50 % RH, 16:8 h photo:scotophase). Neonate larvae were used in the experiments. The solitary parasitoid *M. mediator* (Hymenoptera: Braconidae) was reared on *M. brassicae* feeding on Brussels sprouts in a greenhouse (22 ± 1 °C, 60 ± 10 % RH, 16:8 h photo: scotophase). Parasitoid cocoons were collected and incubated until emergence in a climate cabinet (22 °C, 16:8 h photo:scotophase), supplemented with honey and water. In all experiments, 2- to 7-day-old naive mated female parasitoids were used.

### Rhizobacterium *Pf.* WCS417r growth, inoculation of soil media, and quantification

The rifampicin-resistant, nonpathogenic rhizobacterium strain *Pf.* WCS417r was used in this study. The rhizobacterium was grown for 48 h at 28 °C on King’s B (KB) medium agar plates containing rifampicin (25 µg ml^−1^; Pieterse et al. 1996). Prior to mixing with sterile soil, bacterial cells were collected, re-suspended in 10 mM MgSO_4_, and washed three times with 10 mM MgSO_4._ Afterwards, the bacterial cells were re-suspended in 10 mM MgSO_4_ and adjusted to a cell density of 1 × 10^9^ cfu ml^−1^ (OD^660^ = 1.0). For rhizobacterial treatment, 50 ml of the bacterial suspension were mixed per kg of autoclaved soil; for control treatment, 50 ml of 10 mM MgSO_4_ were mixed per kg of sterile soil. Quantification of *Pf.* WCS417r in *A. thaliana* roots was done for each batch following a well-established method (Pangesti et al. 2015; Pieterse et al. 1998).

### Behavioral test of the parasitoid wasp *M. mediator*

Dual-choice tests were performed using a closed-system Y-tube olfactometer that was illuminated from above (Pineda et al. 2013; Snoeren et al. 2009). Details of the Y-tube olfactometer setup and behavioral tests were similar to the description in Pineda et al. (2013). Experiments were repeated on several days, with ca. 20 female wasps tested per pair-wise comparison per day. In total, 4–5 sets of plants and 88–98 female wasps were evaluated per pair-wise comparison. The plants that were tested as odor sources had been subjected to one of four treatments, based on the presence/absence of rhizobacteria and *M. brassicae* caterpillars: (1) control uninfested (C); (2) rhizobacteria-treated uninfested (R); (3) control infested with *Mamestra* caterpillars (CM); (4) rhizobacteria-treated plants infested with *Mamestra* caterpillars (RM). In the treatments with caterpillars, *A. thaliana* plants (5–6 weeks old) were infested with three neonatal larvae of *M. brassicae* for 3 days before the experiments. Individual plants from all treatments were confined in a plastic container (height 14 cm; upper diameter 11 cm, lower diameter 8.5 cm) covered with insect-proof mesh cloth and sealed with elastic bands. Plants were kept in a growth chamber under 16:8 h photo:scotophase (200 µmol m^−2^ s^−1^) at 21 ± 1 °C and 60–70 % RH. Four plants together comprised an odor source. After the behavioral bioassay, *M. brassicae* larvae from control (CM) and rhizobacteria-treated plants (RM) were recovered and weighed (microbalance CP2P, Sartorius AG, Göttingen, Germany). Additionally, a pool of four plant rosettes of all treatments (C, R, CM, RM) were weighed after each dual-choice assay.

To evaluate if *M. mediator* responds to volatiles from caterpillar-infested *A. thaliana* Col-0 as host-location cues, the following dual-choice experiment was conducted as a control: control uninfested (C) versus control caterpillar-infested plants (CM). To assess whether *M. mediator* responds to volatiles from rhizobacteria-treated caterpillar-infested plants, the following experiment was conducted: rhizobacteria-treated undamaged (R) versus rhizobacteria-treated caterpillar-infested plants (RM). To test the second hypothesis, that the effect of rhizobacteria is a result of the plant’s interaction with both *M. brassicae* and rhizobacteria and not simply a rhizobacteria–plant interaction, the following experiments were conducted: control caterpillar-infested (CM) versus rhizobacteria-treated caterpillar-infested plants (RM); control uninfested (C) versus rhizobacteria-treated uninfested (R).

### Headspace collection and analysis of volatiles

To link parasitoid behavior to volatile emission, collection of plant volatiles was conducted simultaneously with the behavioral assays. In order to correct for background volatiles, volatiles collected from empty jars, empty plant pots, plant pots filled with autoclaved soil, and plant pots filled with autoclaved and rhizobacteria-treated soil were sampled as well. Plant volatiles were collected for 4 h by drawing air out of the jars at a rate of 200 ml min^−1^ with the help of an external pump through a stainless steel cartridge (Markes, Llantrisant, UK) filled with 200 mg Tenax TA (20/35 mesh; CAMSCO, Houston, TX, USA) (Pineda et al. 2013). Immediately after the collection of volatiles, plant rosettes were weighed and the Tenax TA cartridges were dry-purged for 10 min with nitrogen (N_2_, 50 ml min^−1^) at room temparature (RT) and then also stored at RT until analysis. For each treatment, 9–10 replicates were sampled.

Headspace samples were analyzed with a Thermo Trace Ultra gas chromatograph (GC) coupled to a Thermo Trace DSQ quadrupole mass spectrometer (MS), both from Thermo Fisher Scientific (Waltham, MA, USA). Volatiles were desorbed from the cartridges using a thermal desorption system at 250 °C for 10 min (Ultra 50:50, Markes) with a helium flow of 20 ml min^−1^. Analytes were focused at 0 °C on an electronically cooled sorbent trap (Unity, Markes) and then transferred in splitless mode to the analytical column (ZB-5MSi, 30 m × 0.25 mm i.d. 0.25 × mm film thickness with a 5 m built-in guard column; Phenomenex, Torrence, CA, USA) situated in the GC oven for further separation by rapid heating of the cold trap at a rate of 40 °C s^−1^ to 280 °C, which was maintained for 10 min. The GC was held at an initial temperature of 40 °C for 2 min, and then a linear thermal gradient of 10 °C min^−1^ was applied up to 280 °C, where it was held for 4 min, under a column flow of 1 ml min^−1^. The column effluent was ionized by electron impact ionization at 70 eV. Mass spectra were acquired by scanning from *m*/*z* 35 to 350at a scan rate of 5.38 scans s^−1^. The MS transfer line and ion source were set to 275 and 250 °C, respectively. Tentative identification of compounds was made by comparing mass spectra with those in NIST 2005 and the Wageningen Mass Spectral Libraries of Natural Products. Experimentally calculated linear retention indices (LRI) were also used as an additional criterion to identify the compounds. Relative quantification (peak areas of individual compounds) was achieved using a single (target) ion in selected ion monitoring (SIM) mode. The individual peak areas of each compound were further used in the statistical analysis. Volatiles from empty glass jars, empty plant plastic pots, pots filled with autoclaved soil, pots filled with autoclaved and rhizobacteria-treated soil, clean Tenax TA, and the analytical instrument itself were used as a control measure for artefacts.

### Gene transcript analyses of two terpene synthase genes, *TPS03* and *TPS04*

To evaluate if rhizobacterial colonization itself and in combination with insect herbivory by *M. brassicae* has an effect on the transcription of two genes coding for enzymes involved in plant volatile producion, the terpene synthase genes *TPS03* and *TPS04*, the same four treatments as used in the behavioral assay were arranged. Fully expanded leaves of plants exposed to feeding larvae were sampled after gently removing the caterpillars. Leaves were harvested at 10 and 24 h after insect infestation (hpi). Leaves of uninfested plants were treated and harvested at similar time points as those of infested plants. Leaf samples were immediately frozen in liquid nitrogen and stored at −80 °C for RNA extraction. For each treatment, five biological replicates were used, each consisting of 6–9 local leaves pooled from three individual plants. The procedures for processing leaf samples, measuring RNA quality, and synthesizing cDNA synthesis followed the methods described in Pangesti et al. (2015).

Transcripts of the *Terpene Synthase* (*TPS*) gene *TPS03* (AT4G16740) with the sequences of the forward primer 5′-GCCACCATCCTCCGTCTC-3′ and the reverse primer 5′-CCAAGCCACACCGATAATTCC-3′, and the* TPS* gene *TPS04* (AT1G61120) with the sequences of the foward primer 5′-TCGCAGCACACACCATTG-3′ and the reverse primer 5′-GAGCAGCACGGAGTTCATC-3′ (Snoeren et al. 2010) were quantified by qRT-PCR (CFX96™ Real-Time System, BIO-RAD, Hercules, CA, USA). The efficiency of each primer was determined before qRT-PCR analysis. Thermal cycling conditions consisted of 95 °C for 3 min and then 40 cycles at 95 °C for 15 s and at 62 °C for 45 s. For each primer pair, controls without the addition of template were performed to confirm that primer dimers were not interfering with the detection of amplification. The transcript level for each tested gene was calculated relative to the reference genes *ELONGATION FACTOR 1α* (*EF1α*) (AT5G60390) with the sequences of the forward primer 5′-TGAGCACGCTCTTCTTGCTTTCA-3′ and the reverse primer 5′-GGTGGTGGCATCCATCTTGTTACA-3′, and *F*-*BOX FAMILY PROTEIN* (*FBOX*) (AT5G15710) with the sequences of the forward primer 5′-TTTCGGCTGAGAGGTTCGAGT-3′ and the reverse primer 5′-GATTCCAAGACGTAAAGCAGATCAA-3′ (Remans et al. 2008) using the 2^−ΔΔ*Ct*^ method (Livak and Schmittgen 2001).

### Performance of the parasitoid *M. mediator*

To evaluate the effect of rhizobacteria on the development of *M. mediator*, the performance of the parasitoid in its host *M. brassicae*, feeding on either control plants (CM) or rhizobacteria-treated plants (RM), was assessed. Neonatal caterpillars were allowed to feed for 3 days on control plants (C) or rhizobacteria-treated plants (R). One parasitoid female was allowed to parasitize 3 caterpillars reared on control plants (C) and 3 caterpillars reared on rhizobacteria-treated plants (R). Afterwards, these parasitized caterpillars were placed again on plants subjected to the corresponding treatments. All plants were placed individually in plastic containers under the same conditions as described above. Plants were watered three times a week, adding a total of 60 ml of water. On day 8 after infestation, the parasitized caterpillars were transferred to a second plant to avoid food limitation. Survival of parasitized caterpillars and fresh plant biomass were assessed after 8 days of feeding by parasitized caterpillars. Once cocoons were formed, each cocoon was individually kept in a glass tube closed with cotton wool until adult wasps emerged. Cocoon fresh weight was measured 2 days after their formation. Once the adult parasitoids emerged, the sex of each was recorded and then the parasitoids were anesthetized using CO_2_ and weighed on a microbalance to the nearest µg. The following parameters of parasitoid performance were measured: development time from parasitization to cocoon formation; time from cocoon formation to adult emergence (pupal development time) and time from parasitization to adult emergence (total development time); cocoon fresh weight; larval, pupal, and overall survival; fresh weight of male and female parasitoids. In total, 25 plants and 75 parasitized larvae were assessed for each treatment.

### Statistics

Behavioral data were analyzed using a binomial test. After each of the behavioral assays, we assessed the performance of *M. brassicae* on the plants used for the behavioral assays. Data on *M. brassicae* larval weight were analyzed with a linear mixed model (LMM) with treatment as a fixed factor and experimental group (since four plants were placed in the glass jar and larvae would move around during the behavioral test) as a random factor. Data on plant shoot biomass comparing four treatments were analyzed with a two-way ANOVA. Plant volatile data were log-transformed, univariate scaled, and analyzed with multivariate projection to latent structures discrimination analysis (PLS-DA) (SIMCA P+ 12.0, Umetrics AB, Umeå, Sweden). Pair-wise comparisons between treatments of the quantity of each volatile compound emitted were performed with a *t* test. Gene transcription data were log-transformed and analyzed with a two-way ANOVA with treatment and time as factors.

Developmental times of *M. mediator* were analyzed using a generalized linear mixed model (GLMM) with treatment as a fixed factor and plant number as a random factor. The survival data were analyzed using a generalized linear model (GLM) with a binomial distribution and logit link function. Adult fresh weight was analyzed with a *t* test. LSD tests were used for post hoc comparisons when necessary. All data except for the volatile data were analyzed using GenStat 16th edition (VSN International Ltd., Hemel Hempstead, UK).

## Results

### Rhizobacterial colonization enhances attraction of the parasitoid *M. mediator* in plants infested with *M. brassicae*

**Fig. 1:**
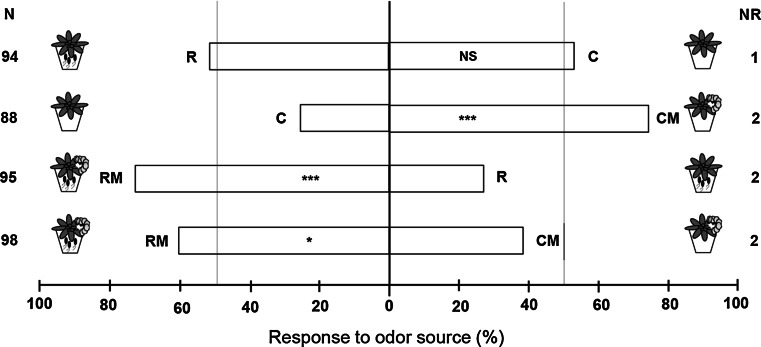
Response of *M. mediator* naïve female parasitoids in a Y-tube olfactometer to the volatiles of *A. thaliana* Col-0 plants from different treatments. Treatments were control plants (*C*), rhizobacteria-treated plants (*R*), control plants infested with *M. brassicae* caterpillars (*CM*), or rhizobacteria-treated plants infested with *M. brassicae* caterpillars (*RM*). Nine to ten sets of plants were used per pair-wise comparison, each consisting of four plants per treatment. *Bars* represent the percentages of parasitoids that choose each of the two odor sources; *N* number of parasitoids that make the choice, *NR* number of nonresponsive parasitoids. *Asterisks* indicate significant differences (binomial test: *P < 0.05; ***P < 0.001; *NS* not significant)

In dual-choice olfactometer assays, the parasitoid wasps did not discriminate between volatiles emitted from control plants (C) and those emitted from rhizobacteria-treated plants (R) (Fig. 1; binomial test, *P* = 0.918). In contrast, the wasps significantly preferred the volatiles emitted from *M. brassicae*-infested plants (CM) over volatiles emitted from control plants (C) (binomial test, *P* < 0.001). Likewise, the parasitoid wasp also significantly preferred the volatiles emitted from rhizobacteria-treated plants infested with *M. brassicae* (RM) over volatiles emitted from rhizobacteria-treated plants (R) (binomial test, *P* < 0.001). Interestingly, rhizobacterial colonization of *A. thaliana* roots significantly increased the parasitoid wasp preference for the volatiles emitted from *M. brassicae*-infested plants (RM) compared to the volatiles emitted from plants without rhizobacteria and infested with *M. brassicae* (CM) (binomial test, *P* = 0.033).

**Fig. 2:**
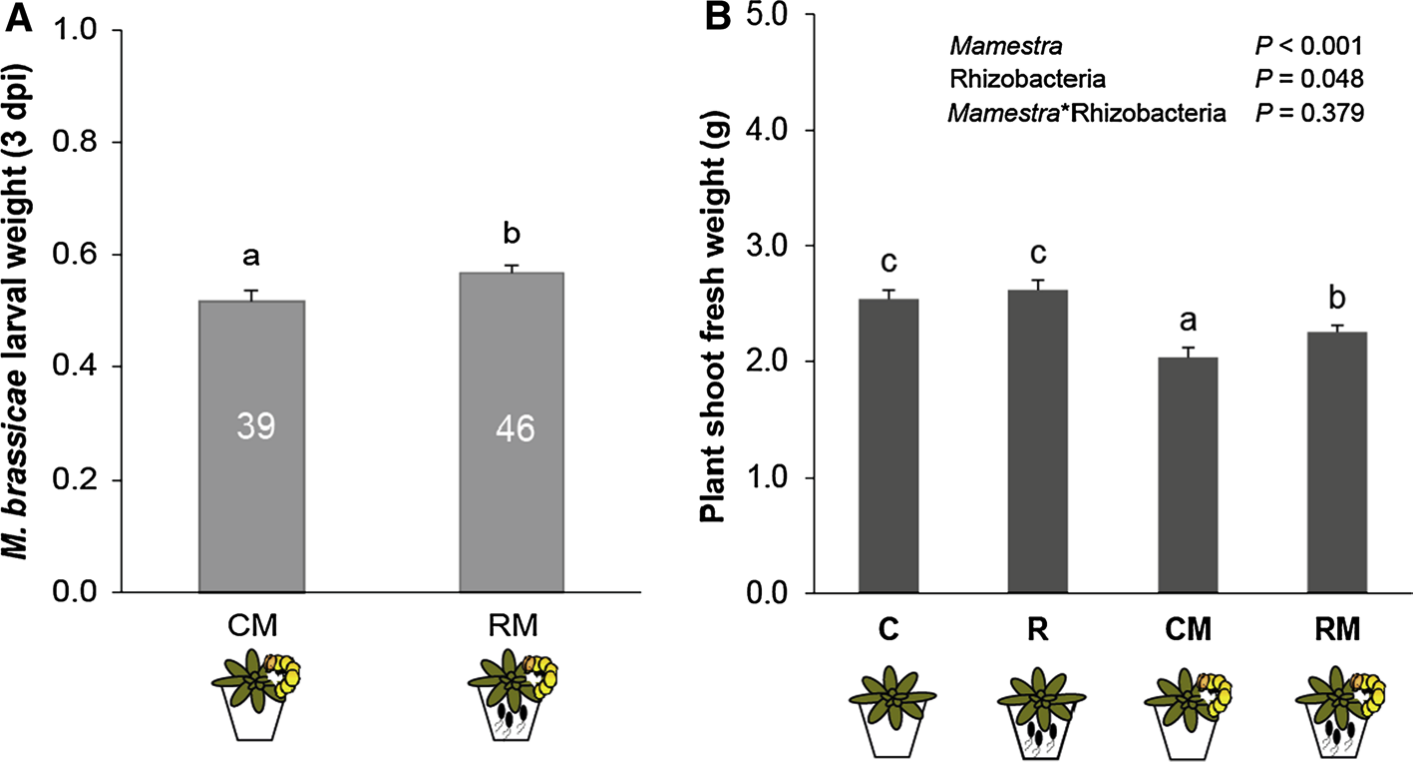
**a** Body mass (mean ± SE) of caterpillars feeding on the sets of plants from the olfactometer assays for 3 days. *CM* control plants, *RM* rhizobacteria-treated plants. Three neonatal larvae were placed on each plant and a set of four plants was used in each experiment. *Numbers* *inside each* *bar* represent the number of larvae surviving on the day of weight assessment. *Different letters* *above* *bars* indicate significant differences between treatments (LMM, *P* < 0.05, LSD test). **b** Plant shoot fresh weight (mean ± SE) of control plants (*C*), rhizobacteria-treated plants (*R*), control (*CM*), or rhizobacteria-treated plants infested with *M. brassicae* for 6 days (*RM*). *Different letters* *above* *bars* indicate a significant difference between treatments (two-way ANOVA, *N* = 12–15 plants, *P* < 0.05, LSD test)

The rhizobacterial treatment resulted in increased larval weight at 3 dpi (Fig. 2a; *df* = 1, 81.9; *F* = 4.29; *P* = 0.042). Assessing plant shoot fresh weight after 6 days of *M. brassicae* feeding showed that both *M. brassicae* feeding and rhizobacterial colonization significantly affected plant biomass, but there was no interaction between the two factors (two-way ANOVA, *Mamestra*: *df* = 1, 53; *F* = 31.76; *P* < 0.001; rhizobacteria: *df* = 1, 53; *F* = 4.12; *P* = 0.048; *Mamestra* × rhizobacteria: *df* = 1, 53; *F* = 0.79; *P* = 0.379) (Fig. 2b). Rhizobacterial colonization of *A. thaliana* Col-0 roots (R) had no effect on the shoot fresh weight in comparison to control plants (C). Interestingly, under caterpillar attack, rhizobacteria-treated plants (RM) grew better than control plants infested with caterpillars (CM). Whereas plant shoot fresh weight after 3 days of *M. brassicae* feeding showed that herbivory significantly affected plant biomass, rhizobacteria had no effect on plant biomass (Fig. S1 of the Electronic supplementary material, ESM; two-way ANOVA, *Mamestra*: *df* = 1, 37; *F* = 5.75; *P* = 0.022; rhizobacteria: *df* = 1, 37; *F* = 1.72; *P* = 0.199; *Mamestra* × rhizobacteria: *df* = 1, 37; *F* = 0.41; *P* = 0.525). No rifampicin-resistant rhizobacteria were detected in the rhizosphere of control plants (detection limit 10^2^ cfu g^−1^), whereas the numbers of rhizobacteria in the rhizosphere of rhizobacteria-treated plants were overall >10^5^ cfu g^−1^ roots (Table S1 of the ESM).

### Herbivory by *M. brassicae* increases volatile emission of control and rhizobacteria-treated *A. thaliana* plants

**Fig. 3:**
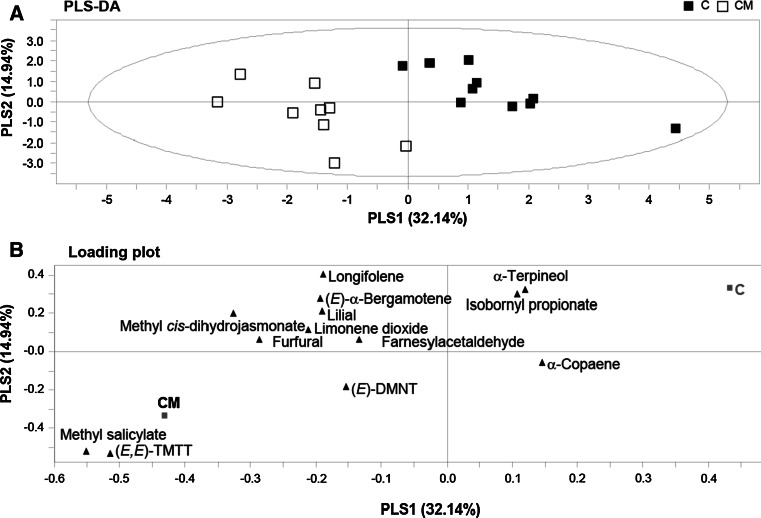
Projection to latent structures discriminant analysis (PLS-DA) comparing volatile blends from control plants (*C*) versus control plants infested with *Mamestra brassicae* (*CM*) for 3 days before volatile collection. **a** Grouping pattern of samples according to the first two principal components and Hotelling’s ellipse of the 95 % confidence interval for the observations. Each* point *represents one sample (*N* = 9–10 replicates). **b** Loading plot of the first two components of the PLS-DA, showing the contribution of each volatile compound to the separation of the two treatments

A PLS-DA analysis of control plants (C) and control plants infested with *M. brassicae* (CM) showed four significant principal components (PC), with the first two explaining 32.14 and 14.94 % of the total variance, respectively (Fig. 3a). The first component (PLS 1) separated the volatile blends based on the presence or absence of *M. brassicae* caterpillars. A total of 13 compounds were detected in the headspace, and 3 compounds had a VIP value higher than 1. VIP values indicate the importance of the variable, i.e., the volatile compound, in the projection, and those with values larger than 1 are the most influential in the model (Eriksson et al. 2006). In decreasing order of importance, these compounds were methyl salicylate, (*E*,*E*)-TMTT, and methyl *cis*-dihydrojasmonate (Fig. 3b). These three compounds were emitted in significantly higher amounts by CM than by C plants (Table S2 of the ESM; *t* test; *P* < 0.05). All other compounds were emitted in statistically similar quantities from CM and C plants (Table S2 of the ESM; *t* test; *P* > 0.05).

**Fig. 4:**
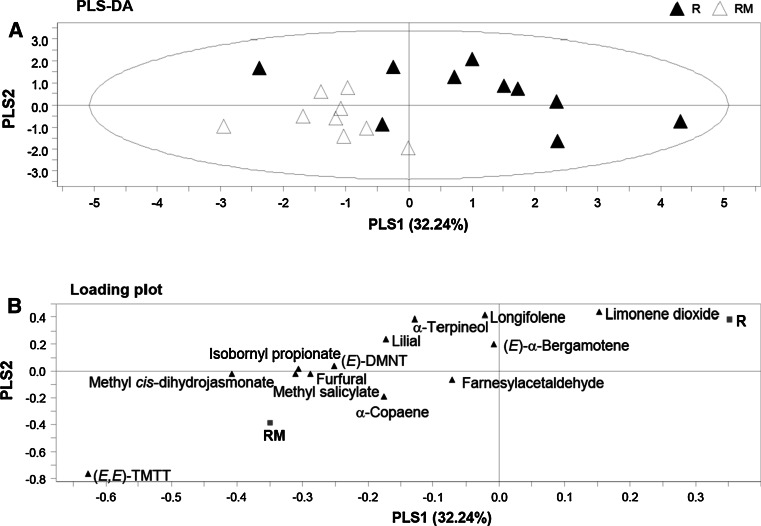
Projection to latent structures discriminant analysis (PLS-DA) comparing volatile blends from rhizobacteria-treated (*R*) versus rhizobacteria-treated plants infested with *M. brassicae* (*RM*) for 3 days before volatile collection. **a** Grouping pattern of samples according to the first two principal components and Hotelling’s ellipse of the 95 % confidence interval for the observations. Each *point* represents one sample (*N* = 9–10 replicates). **b** Loading plot of the first two components of the PLS-DA, showing the contribution of each volatile compound to the separation of the two treatments

A PLS-DA of rhizobacteria-treated plants (R) and rhizobacteria-treated plants infested with *M. brassicae* (RM) showed one significant PC explaining 32.24 % of the total variance (Fig. 4a). The second axis is shown for representational purposes. This PC also separated the volatile blends based on the presence or absence of caterpillar *M. brassicae*. Of the 13 compounds recorded, 3 compounds showed a VIP value higher than 1. In decreasing order of importance, these compounds were (*E*,*E*)-TMTT, methyl *cis*-dihydrojasmonate, and α-terpineol (Fig. 4b). (*E*,*E*)-TMTT and (*E*)-α-bergamotene were emitted in significantly higher amounts by RM than by R plants (Table S2 of the ESM; *t* test; *P* < 0.05), whereas the emission rates of all other compounds were statistically similar in RM and R plants.

When all four treatments of control plants (C), rhizobacteria-treated plants (R), control plants infested with *M. brassicae* (CM) and rhizobacteria-treated plants infested with *M. brassicae* (RM) were analyzed together in one PLS-DA, it gave one significant PC explaining 26.09 % of the total variance. Similar to the PLS-DA comparing C-CM and R-RM, this PC separated the volatile blends based on the presence or absence of caterpillar *M. brassicae*. Among the 13 compounds recorded, 4 compounds had VIP values higher than 1. In decreasing order of importance, these compounds were (*E*,*E*)-TMTT, methyl salicylate, methyl *cis*-dihydrojasmonate, and lilial (Fig. S2 of the ESM).

### Rhizobacterial colonization suppresses volatile emission of *A. thaliana* following *M. brassicae* herbivory

**Fig. 5:**
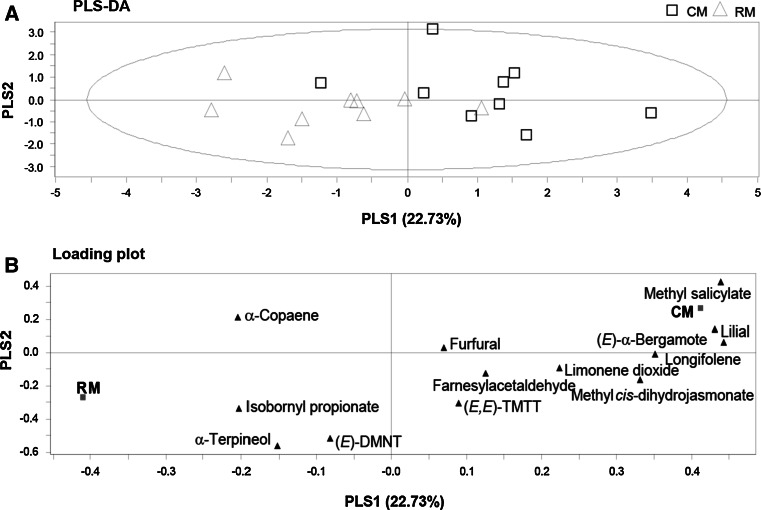
Projection to latent structures discriminant analysis (PLS-DA) comparing volatile blends from either control (*CM*) or rhizobacteria-treated (*RM*) plants infested with *M. brassicae* for 3 days before volatile collection. **a** Grouping pattern of samples according to the first two principal components and Hotelling’s ellipse of the 95 % confidence interval for the observations. Each* point* represents one sample (*N* = 9–10 replicates). **b** Loading plot of the first two components of the PLS-DA, showing the contribution of each volatile compound to the separation of the two treatments

A PLS-DA comparing the volatiles emitted by control plants infested with *M. brassicae* (CM) and rhizobacteria-treated plants infested with *M. brassicae* (RM) showed one significant PC explaining 22.73 % of the total variance (Fig. 5a). The second axis is shown for representational purposes. This PC separated the volatiles based on the presence or absence of the rhizobacteria. Five compounds had a VIP value higher than 1, and these were, in decreasing order of importance, methyl salicylate, (*E*)-α-bergamotene, lilial, longifolene, and methyl *cis*-dihydrojasmonate (Fig. 5b). Among these five compounds, methyl salicylate, (*E*)-α-bergamotene, and lilial were emitted in significantly lower amounts by RM than by CM plants (Table S2 of the ESM; *t* test; *P* < 0.05), whereas emission rates of all other compounds were statistically similar in RM and CM plants. Interestingly, a PLS-DA comparing volatiles of control (C) and rhizobacteria-treated plants (R) showed no significant principal component. Pair-wise comparisons for the quantities emitted of each of the 13 compounds detected in the two treatments did not show differences (Table S2 of the ESM; *t* test; *P* > 0.05).

### Rhizobacterial colonization modifies transcription of terpene synthase genes *TPS03* and *TPS04*

Transcript analyses of *TPS03* and *TPS04* showed that these genes were affected by both treatment and time (two-way ANOVA, effect of treatment on *TPS03* expression: *df* = 3, 32; *F* = 63.11, *P* < 0.001; time: *df* = 1, 32; *F* = 8.44, *P* < 0.007; effect of treatment on *TPS04* expression: *df* = 3, 32; *F* = 113.96, *P* < 0.001; time: *df* = 1, 32; *F* = 4.68, *P* = 0.038). However, there was no interaction between treatment and time for either *TPS03* or *TPS04* expression.

**Fig. 6:**
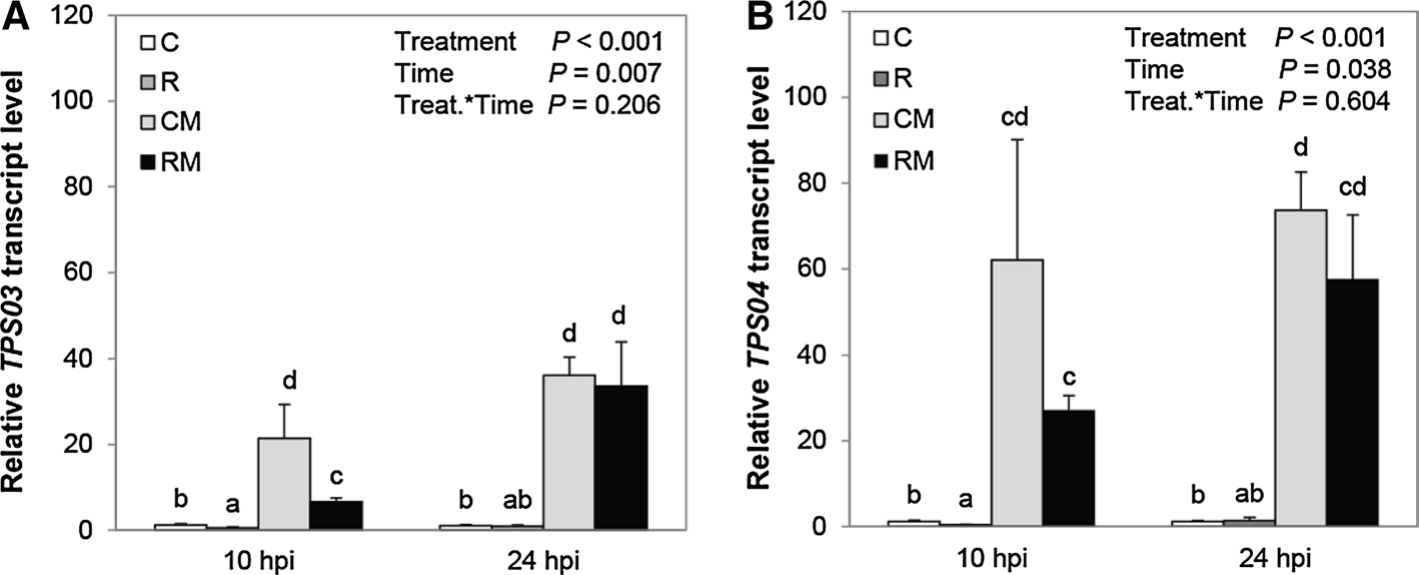
Relative transcript levels (mean ± SE) of *TPS03* and *TPS04* in local leaves of control plants (*C*), rhizobacteria-treated plants (*R*), control plants infested with *M. brassicae* (*CM*), or rhizobacteria-treated plants infested with *M. brassicae* (*RM*) for 10 and 24 h post-infestation (hpi). Transcript levels were normalized relative to the reference genes *EF1α* and *FBOX* and measured relative to the control plants (*N* = 5 replicates, each from a pool of 3 plants). *Different letters* *above* *bars* indicate a significant difference between treatments (two-way ANOVA, *P* < 0.05, LSD test)

At 10 hpi, rhizobacterial colonization (R) resulted in a downregulation of both *TPS03* and *TPS04* transcription in comparison to control plants (C) (Fig. 6a, b); however, at 24 hpi, gene expression did not differ between treatments (two-way ANOVA, *P* > 0.05, LSD test). At both time points, feeding damage caused by *M. brassicae* (CM) resulted in a significantly increased expression of *TPS03* and *TPS04* in comparison to control plants (C). Rhizobacteria-treated plants infested with *M. brassicae* (RM) showed in significantly higher expression of *TPS03* and *TPS04* when compared to uninfested plants (R). Contrary to our hypothesis, at 10 hpi, the expression of *TPS03* in rhizobacteria-treated plants infested with *M. brassicae* (RM) was significantly lower than that in control plants infested with *M. brassicae* (CM). A lower mean expression level was also found for *TPS04*, but the difference was not significant. Interestingly, after 24 h of feeding by *M. brassicae* on rhizobacteria-treated plants (RM), the expression of *TPS03* significantly increased to a level similar to that seen for control plants infested with *M. brassicae* (CM). A similar pattern was also observed in the expression of *TPS04*.

### Rhizobacterial colonization does not affect the performance of the parasitoid *Microplitis mediator*

**Fig. 7:**
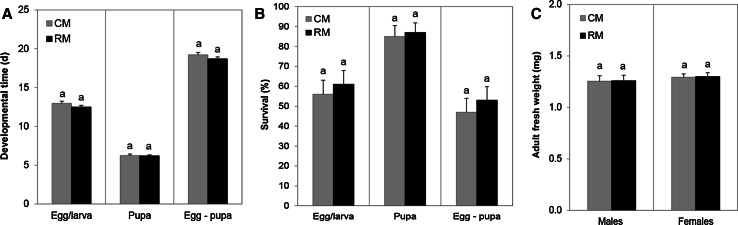
Performance parameters of the parasitoid wasp *M. mediator* developing in its host *M. brassicae*, which is feeding on either control (*CM*) or rhizobacteria-treated (*RM*) plants. Three parasitized larvae were placed on each plant (*N* = 25 plants). Effects of rhizobacterial colonization on **a** developmental times of parasitoid egg/larva; pupa; egg–pupa (GLMM), **b** survival rates of parasitoid egg/larva; pupa; egg–pupa (GLMM), and **c** fresh weights of adult parasitoid males and females (*t* test). *Different letters* *above* *bars* indicate a significant difference between treatments (*P* < 0.05)

Rhizobacterial colonization had no effect on performance parameters of the parasitoid *M. mediator* (Fig. 7a): developmental time of egg/larva (GLMM; *df* = 1, 48.2; Wald stat. = 2.41; *P* = 0.127), pupa (GLMM; *df* = 1, 148; Wald stat. = 0.21; *P* = 0.65;) and egg–pupa (GLMM; *df* = 1, 48; Wald stat. = 1.25; *P* = 0.27). Rhizobacterial colonization had no effect on the survival of egg/larva (GLM, *df* = 1, 49; Wald stat. = 0.29; *P* = 0.593) and pupa (GLM, *df* = 1, 43; Wald stat. = 0.07 *P* = 0.789), or on survival during development from egg to pupa (GLM, *df* = 1, 48; Wald stat. = 0.39; *P* = 0.534) (Fig. 7b). Rhizobacterial treatment also did not affect the fresh weight of *M. mediator* adult males (*t* test, *df* = 1, *F* = 0.023, *P* = 0.964) or females (*t* test *df* = 1, *F* = 0.017, *P* = 0.904) (Fig. 7c).

## Discussion

Our study shows that *P. fluorescens* WCS417r colonization of *A. thaliana* roots results in an increased attraction of the parasitoid wasp *M. mediator* to host-infested plants, but also an increase in larval weight of the herbivore. We previously reported that the effect of rhizobacteria on direct plant defense against *M. brassicae* was variable and that soil nutrient level influenced the strength of this direct defense (Pangesti et al. 2015). Interestingly, when we incorporated a natural enemy of the herbivore into the study system, the effect of rhizobacteria on indirect plant defenses against the herbivore was found to be a consistent increase in parasitoid attraction. Previous studies likewise found that, in general, different groups of beneficial microbes positively affected the attraction of natural enemies of arthropods in different plant species (Battaglia et al. 2013; Guerrieri et al. 2004; Schausberger et al. 2012). For instance, inoculation of bean plants (*Phaseolus vulgaris* L.) with the mycorrhiza *Glomus mosseae* changed the composition of HIPVs after a spider-mite attack, thus increasing the attraction of predatory mites to the spider-mite-infested plants (Schausberger et al. 2012). The effect of mycorrhizal fungi on herbivore parasitization was shown to depend on the mycorrhizal species (Gange et al. 2003). A recent study performed using the same rhizobacteria–plant system as employed in the present study demonstrated that the parasitoid *Diaeretiella rapae* was, however, less attracted to rhizobacteria-inoculated plants infested by its host, the aphid *Myzus persicae*, than to host-infested plants without rhizobacteria (Pineda et al. 2013). Therefore, our present study, together with Pineda et al. (2013), shows that the effect of a certain beneficial microbe on indirect plant defense depends on the particular species of insect herbivores and their parasitoids considered.

Volatile analysis of control and rhizobacteria-treated plants infested with *M. brassicae* (CM versus RM) showed that both treatments had quantitative but not qualitative differences. In the absence of the herbivore, rhizobacteria themselves did not emit or induce volatiles that affect the searching behavior of the parasitoid. In contrast to our hypothesis, instead of an increased emission of HIPVs, rhizobacterial colonization of *A. thaliana* roots reduced the emission of several HIPVs. The rhizobacteria-treated plants infested with caterpillars (RM) emitted lower amounts of the aromatics methyl salicylate, lilial, and the terpene (*E*)-α-bergamotene in comparison to control plants infested with caterpillars (CM). The gene transcriptional results correlate with these results, showing a trend for an overall negative effect of rhizobacteria colonization on the expression levels of the terpene synthase genes *TPS03* and *TPS04*. In line with these findings, a previous study on *Plantago lanceolata* plants found that mycorrhizal (*Rhizophagus irregularis*, formerly known as *Glomus intraradices*) colonization suppressed the emission of several terpenoids by plants infested with the caterpillar *Spodoptera exigua* (Fontana et al. 2009). Whether mycorrhiza-induced suppression of HIPVs affects the behavior of natural enemies of herbivorous insects has not been evaluated, since most studies have focused either on the emission of VOCs or on the effects on the parasitoids. In our previous study with rhizobacteria and aphids, we saw the opposite pattern: decreased attraction of aphid parasitoids to rhizobacteria-treated host-infested plants was associated with increased emission of several HIPVs (Pineda et al. 2013). These examples indicate that microbe-induced emission of HIPVs does not necessarily translate to increased attractiveness of parasitoids, and that the blend composition likely plays an important role (Wijk et al. 2011).

Plants face an important dilemma—whether to allocate resources to growth or defense (Herms and Mattson 1992)—and it is likely that the interaction with beneficial microbes belowground could help plants to accommodate both strategies (Bennett et al. 2006; Pangesti et al. 2013). Our data show that rhizobacterial colonization resulted in an increase in plant growth in the presence of caterpillars, but no increased growth of uninfested plants, and this effect was only significant after longer caterpillar infestation. The rhizobacterium *P. fluorescens* WCS417r is known to promote root growth by modulating auxin signaling in the plant (Zamioudis et al. 2013), which could increase plant access to soil nutrients and enhance plant growth. Additionally, this rhizobacterial strain increased soluble carbohydrates in tomato plants (Shavit et al. 2013). Evidence from other microbes has shown that, for instance, mycorrhizal fungi can also increase nitrogen foliar content (Azcon et al. 1991; Gange et al. 2005). From the herbivore’s perspective, increased levels of these plant nutrients can enrich their diet and consequently increase herbivore performance (Mattson 1980; Roeder and Behmer 2014), supporting our observations of increased *M. brassicae* performance in rhizobacteria-colonized plants. However, an investigation of why rhizobacteria-induced growth promotion only occurs under herbivore pressure is needed. Interestingly, two studies on cotton (*Gossypium hirsutum* L.) and lima bean plants (*Phaseolus lunatus* L.) showed that increased nitrogen fertilization suppressed the synthesis of various terpenoids by *S. exigua*-induced plants (Ballhorn et al. 2013; Chen et al. 2008). We hypothesize that, in our study system, *P. fluorescens* WCS417r led to an increase in the levels of nitrogen or other mineral nutrients in the plants, thus suppressing the emission of terpenoids and aromatic volatiles following caterpillar herbivory. The synthesis of volatile terpenoids is regulated by the JA signaling pathway (Dicke and Poecke 2002; Maffei et al. 2011; Poecke and Dicke 2002), and JA is also the main plant hormone regulating the switch from growth to defense (Bennett et al. 2006; Pangesti et al. 2013). As shown in this study, rhizobacteria also play an important role in plant defense and growth, but whether JA signaling regulates a rhizobacteria-induced trade-off between growth and synthesis of HIPVs requires further research.

Despite the importance of including the third trophic level to understand plant–herbivore interactions, the effects of beneficial microbes on parasitoids and predators—along with the corresponding underlying mechanisms—have only recently begun to be understood (Bennett 2012). By working with the same rhizobacteria–plant system but with herbivores of different feeding guilds (phloem feeders versus leaf chewers), the current study—together with Pineda et al. (2013)—provides new evidence that (1) the effect of rhizobacteria on parasitoid attraction depends on the herbivore–parasitoid complex; (2) changes in the blend composition rather than higher emissions of total volatiles seem to be responsible for these effects; (3) a stronger JA response in the plant triggered by rhizobacteria colonization (Pangesti et al. 2015; Pineda et al. 2012; Oosten et al. 2008) does not correlate with a higher emission of HIPV. Using different biological systems, the few studies available as well as this study (Fontana et al. 2009; Pineda et al. 2013; Schausberger et al. 2012) suggest a pattern whereby root colonization by beneficial microbes decreases the emission of volatile terpenoids following attack by caterpillars, but increases the emission of volatile terpenoids following attack by cell-content feeders (e.g., spider mites) and phloem feeders (e.g., aphids). However, more studies are needed to confirm this pattern, and—in particular—to elucidate the ecological consequences of the attractions of these different types of natural enemies (generalist versus specialist parasitoids). Rhizobacteria affect plants and their associated organisms, so measuring both plant-defense and plant-growth parameters is crucial when attempting to determine if rhizobacterial colonization can indeed encourage plants to allocate extra resources to both growth and defense.

### **Author contribution statement**

NP, AP, JJAvL, MD conceived the study and wrote the manuscript; other authors provided additional input. NP, AP designed and performed experiments. BTW performed volatile analysis. NP, BTW analyzed the data. BL performed some of the experiments.

## Electronic supplementary material

Below is the link to the electronic supplementary material.

Supplementary material 1 (DOCX 139 kb)
