# Supplementary material for: Rhizobacterial colonization of roots modulates plant volatile emission and enhances the attraction of a parasitoid wasp to host-infested plants
Source: Oecologia. 2015 Mar 19;178(4):1169–80. doi: 10.1007/s00442-015-3277-7 (PMC4506461; doi:10.1007/s00442-015-3277-7)
Supplement: Supplementary file 1 — Supplementary material 1 (DOCX 139 kb) [file 442_2015_3277_MOESM1_ESM.docx]

**Supplemental information to:**

**Rhizobacterial colonization of roots modulates plant volatile emission and enhances attraction of a parasitoid wasp to host-infested plants**

Nurmi Pangesti, Berhane T. Weldegergis, Benjamin Langendorf, Joop J.A. van Loon, Marcel Dicke, Ana Pineda

Laboratory of Entomology, P.O. Box 8031, 6700 EH Wageningen, The Netherlands

Corresponding author: Nurmi Pangesti ([nurmi.pangesti@wur.nl](mailto:nurmi.pangesti@wur.nl), npangesti001@gmail.com)

Phone: +31 317 482325

**
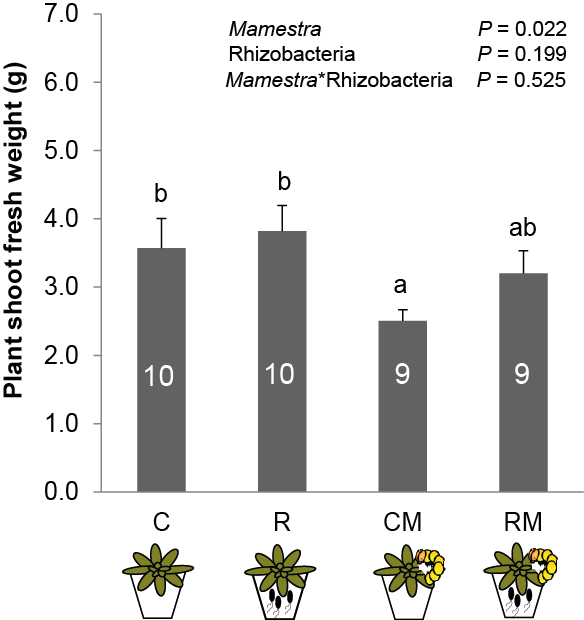
**

**Figure S1.** Shoot fresh weight of *A. thaliana* Col-0, for control plants (C), rhizobacteria-treated plants (R), control plants infested with *M. brassicae* (CM) and rhizobacteria-treated plants infested with *M. brassicae* (RM). The insect herbivore *M. brassicae* were feeding on the plants for 3 days. Data shown are means (± SE) of 4 pooled plant rosettes (two-way ANOVA, *P* < 0.05, LSD test; *N* = 9 -10). Different letters above bars indicate significant difference between treatments (*P* < 0.05).


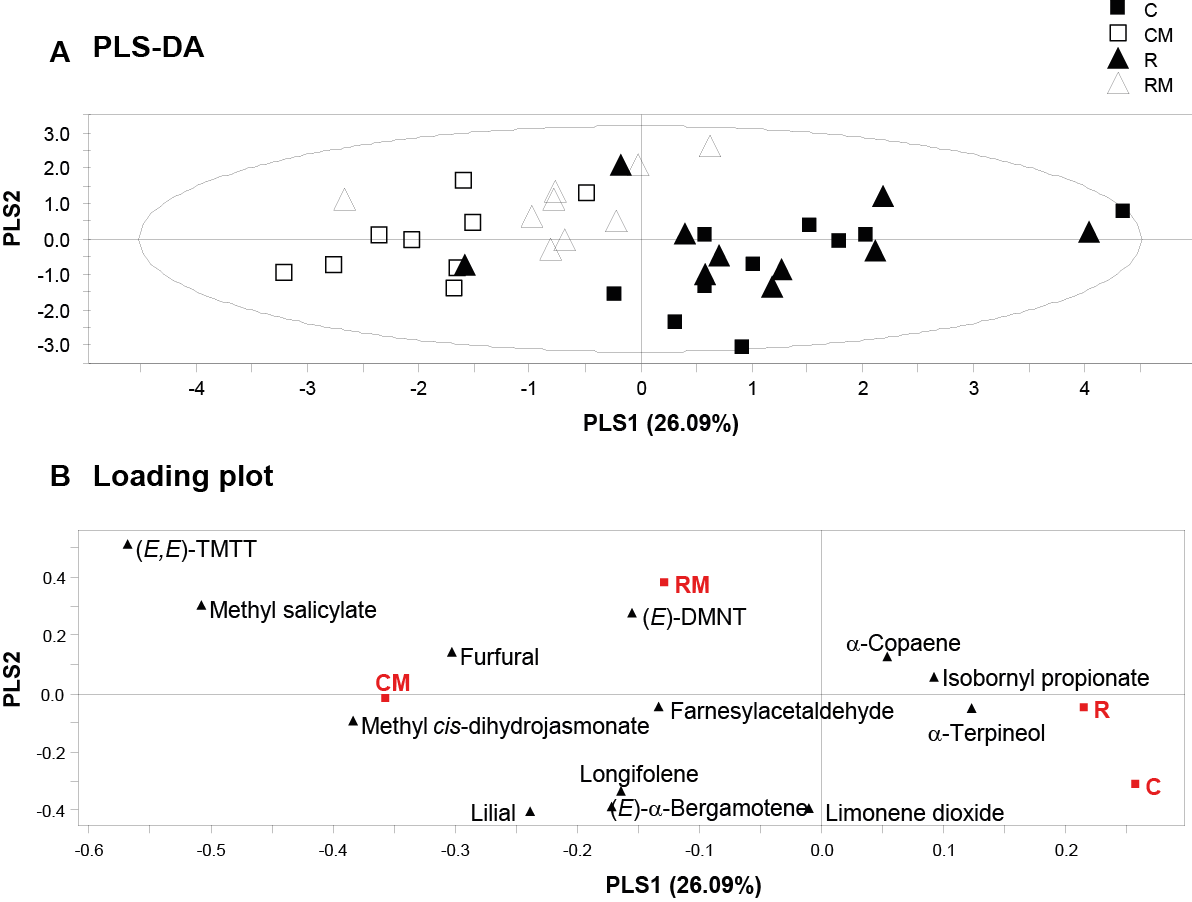


**Figure S2.** Projection to Latent Structures Discriminant Analysis (PLS-DA) comparing *A. thaliana* Col-0 volatile blends from control plants (C), rhizobacteria-treated plants (R), control plants infested with *M. brassicae* (CM), rhizobacteria-treated plants infested with *M. brassicae* (RM). **(A)** Grouping pattern of samples according to the first two principal components and the Hotelling’s ellipse of the 95% confidence interval for the observations. Each point represents one sample (*N* = 9 – 10 replicates). **(B)** Loading plot of the first two components of PLS-DA, showing contribution of each volatile compound to the separation of the four treatments.

| **Table S1:** Rhizobacterial colonization level in different plant batches used in this experiment | | | |
| --- | --- | --- | --- |
|  |  |  |  |
| Treatments | Batch | *N* (plant) | Colony Forming Unit (CFU) g^-1^  of roots |
| Control plants | 1 | 3 | < 100 |
| Rhizobacteria-treated plants | 1 | 3 | 1.7*10^5^ |
|  |  |  |  |
| Control plants | 2 | 2 | < 100 |
| Rhizobacteria-treated plants | 2 | 2 | 1.6*10^5^ |
|  |  |  |  |
| Control plants | 3 | 2 | < 100 |
| Rhizobacteria-treated plants | 3 | 2 | 1.6*10^5^ |
|  |  |  |  |
| Control plants | 4 | 2 | < 100 |
| Rhizobacteria-treated plants | 4 | 2 | 8.6*10^4^ |
|  |  |  |  |
| Control plants | 5 | 3 | < 100 |
| Rhizobacteria-treated plants | 5 | 3 | 5.9*10^5^ |

| **Table S2:** List of volatile organic compounds (VOCs) emitted by control plants (C), rhizobacteria treated plants (R), control plants infested with *Mamestra brassicae* (CM) and rhizobacteria-treated infested with *M. brassicae* (RM) | | | | | | | | | | | | | | | | | | | | | | | | | |
| --- | --- | --- | --- | --- | --- | --- | --- | --- | --- | --- | --- | --- | --- | --- | --- | --- | --- | --- | --- | --- | --- | --- | --- | --- | --- |
|  |  |  |  |  |  |  |  |  |  |  |  |  |  |  | |  | |  | | |  |  | |  |  |
| **No.** | **Compound** |  | **VIP** |  | **Volatile emissions are given as mean (± SE) of GC peak area divided by 10^5^** | | | | | | | | **pairwise comparisons (t-test)** | | | | | | | | | | | | |
|  |  |  |  |  | **C** | | **R** | | **CM** | | **RM** | | **RM vs CM** | | | | **C vs CM** | | | **R vs RM** | | | **R vs C** | | |
|  |  | **C-CM** | **R-RM** | **CM-RM** | **mean** | **SE** | **mean** | **SE** | **mean** | **SE** | **mean** | **SE** | **t** | | **P** | | **t** | | **P** | **t** | | **P** | **t** | | **P** |
|  | ***Terpenoids*** |  |  |  | |  |  |  |  |  |  |  | | |  | |  | |  |  | |  |  | |  |
| **1** | (*E*)-DMNT | 0.57 | 0.81 | 0.77 | 3.17 | 0.64 | 3.14 | 0.46 | 4.68 | 1.35 | 5.47 | 1.54 | -0.36 | | 0.72 | | -1.04 | | -1.04 | -1.29 | | 0.22 | -0.89 | | 0.38 |
| **2** | α-Terpineol | 0.72 | **1.02** | 0.91 | 1.25 | 0.18 | 1.00 | 0.18 | 1.36 | 0.19 | 1.03 | 0.09 | -0.68 | | 0.504 | | 0.81 | | 0.43 | -0.64 | | 0.53 | 0.97 | | 0.34 |
| **3** | Limonene dioxide | 0.78 | 0.89 | 0.77 | 1.98 | 0.46 | 1.98 | 0.37 | 3.44 | 0.97 | 2.65 | 0.70 | 1.02 | | 0.32 | | -1.49 | | 0.16 | 0.76 | | 0.46 | -0.12 | | 0.91 |
| **4** | Isobornyl propionate | 0.54 | 0.96 | 0.78 | 1.17 | 0.28 | 1.08 | 0.30 | 1.62 | 0.56 | 1.32 | 0.13 | -0.93 | | 0.37 | | 0.72 | | 0.48 | -1.61 | | 0.13 | 0.46 | | 0.65 |
| **5** | α-Copaene | 0.61 | 0.56 | 0.78 | 0.50 | 0.06 | 0.49 | 0.10 | 0.46 | 0.08 | 0.49 | 0.08 | -0.93 | | 0.37 | | 0.99 | | 0.34 | -0.88 | | 0.39 | 0.94 | | 0.36 |
| **6** | Longifolene | 0.98 | 0.92 | **1.17** | 2.32 | 0.25 | 2.28 | 0.29 | 2.78 | 0.29 | 2.21 | 0.17 | 1.69 | | 0.11 | | -1.30 | | 0.21 | -0.11 | | 0.91 | 0.21 | | 0.84 |
| **7** | (*E*)-α-Bergamotene | 0.85 | 0.43 | **1.46** | 1.11 | 0.15 | 1.01 | 0.13 | 1.36 | 0.14 | 0.96 | 0.10 | 2.25 | | **0.04** | | -1.34 | | 0.20 | -0.04 | | 0.97 | 0.59 | | 0.57 |
| **8** | (*E*,*E*)-TMTT | **1.70** | **2.07** | 0.57 | 8.09 | 1.40 | 8.86 | 2.01 | 41.09 | 9.32 | 38.97 | 12.89 | 0.40 | | 0.69 | | -5.95 | | **<0.001** | -4.59 | | **<0.001** | -0.09 | | 0.93 |
| **9** | Farnesylacetaldehyde | 0.54 | 0.22 | 0.48 | 163.62 | 61.95 | 129.63 | 44.47 | 255.54 | 93.21 | 158.90 | 46.77 | 0.56 | | 0.58 | | -0.90 | | 0.38 | -0.35 | | 0.73 | -0.03 | | 0.97 |
|  | Total terpenoids |  |  |  | 183.21 | 61.70 | 149.47 | 44.11 | 312.32 | 91.66 | 212.01 | 45.26 | .879 | | .392 | | -1.786 | | .092 | -1.334 | | .200 | .036 | | .972 |
|  | ***Aromatics*** |  |  |  | |  |  |  |  |  |  |  | | |  | |  | |  |  | |  |  | |  |
| **10** | Methyl salicylate | **1.78** | 0.94 | **1.51** | 1.82 | 0.25 | 5.57 | 2.35 | 11.29 | 2.66 | 6.30 | 1.07 | 2.23 | | **0.04** | | -7.62 | | **<0.001** | -1.63 | | 0.12 | -1.50 | | 0.15 |
| **11** | Lilial | 0.75 | 0.85 | **1.42** | 5.96 | 1.65 | 3.61 | 0.59 | 8.73 | 2.04 | 4.14 | 0.63 | 2.18 | | **0.04** | | -1.31 | | 0.21 | -0.86 | | 0.40 | 0.99 | | 0.34 |
|  | Total aromatics |  |  |  | 7.78 | 1.67 | 9.18 | 2.34 | 20.02 | 2.62 | 10.44 | 1.61 | 3.64 | | **.002** | | -4.18 | | **.001** | -1.139 | | .270 | -.358 | | .725 |
|  | ***Others*** |  |  |  | |  |  |  |  |  |  |  | | |  | |  | |  |  | |  |  | |  |
| **12** | Furfural | 0.98 | 0.88 | 0.23 | 11.67 | 1.15 | 13.33 | 1.75 | 21.23 | 4.43 | 18.05 | 2.61 | 0.31 | | 0.76 | | -2.12 | | **0.05** | -1.50 | | 0.15 | -0.52 | | 0.61 |
| **13** | Methyl *cis*-dihydrojasmonate | **1.16** | **1.25** | **1.16** | 9.86 | 1.64 | 8.29 | 2.17 | 18.34 | 2.26 | 13.21 | 1.91 | 1.58 | | 0.13 | | -5.95 | | **0.02** | -2.28 | | **0.04** | 0.66 | | 0.52 |
|  | Total others |  |  |  | 21.53 | 2.51 | 21.62 | 3.16 | 39.57 | 5.59 | 31.26 | 3.81 | 1.019 | | .323 | | -2.770 | | **.013** | -1.993 | | .063 | .097 | | .924 |
|  | **Total** |  |  |  | 212.52 | 61.68 | 180.27 | 46.03 | 371.91 | 94.78 | 253.71 | 45.84 | 1.09 | | 0.29 | | -1.99 | | 0.06 | -1.45 | | 0.17 | 0.10 | | 0.92 |
|  |  |  |  |  |  |  |  |  |  |  |  |  |  | |  | |  | |  |  | |  |  | |  |

- VIP value > 1 written in bold.
- *P* < 0.05 written in bold.
